# Triage for coronary artery bypass graft surgery in Canada: Do patients agree on who should come first?

**DOI:** 10.1186/1472-6963-7-118

**Published:** 2007-07-25

**Authors:** Katy Shufelt, Alice Chong, David A Alter

**Affiliations:** 1 Institute of Clinical Evaluative Sciences, Toronto, Canada; 2 Schulich Heart Program of Sunnybrook Health Sciences Centre, Toronto, Canada; 3 Clinical Epidemiology Unit of Sunnybrook Health Sciences Centre, Toronto, Canada; 4 Li Ka Shing Knowledge Institute of St. Michael's Hospital, Toronto, Canada; 5 Division of Cardiology, St. Michaels' Hospital, Toronto, Canada; 6 Department of Medicine, University of Toronto, Toronto, Canada; 7 The Toronto Rehabilitation Institute, Toronto, Canada; 8 Department of Health Policy, Management and Evaluation, University of Toronto, Toronto, Canada

## Abstract

**Background:**

The extent to which clinical and non-clinical factors impact on the waiting-list prioritization preferences of patients in the queue is unknown. Using a series of hypothetical scenarios, the objective of this study was to examine the extent to which clinical and non-clinical factors impacted on how patients would prioritize others relative to themselves in the coronary artery bypass surgical queue.

**Methods:**

Ninety-one consecutive eligible patients awaiting coronary artery bypass grafting surgery at Sunnybrook Health Sciences Centre (median waiting-time duration prior to survey of 8 weeks) were given a self-administered survey consisting of nine scenarios in which clinical and non-clinical characteristic profiles of hypothetical patients (also awaiting coronary artery bypass surgery) were varied. For each scenario, patients were asked where in the queue such hypothetical patients should be placed relative to themselves.

**Results:**

The eligible response rate was 65% (59/91). Most respondents put themselves marginally ahead of a hypothetical patient with identical clinical and non-clinical characteristics as themselves. There was a strong tendency for respondents to place patients of higher clinical acuity ahead of themselves in the queue (P < 0.0001). Social independence among young individuals was a positively valued attribute (P < 0.0001), but neither age per se nor financial status, directly impacted on patients waiting-list priority preferences.

**Conclusion:**

While patient perceptions generally reaffirmed a bypass surgical triage process based on principals of equity and clinical acuity, the valuation of social independence may justify further debate with regard to the inclusion of non-clinical factors in waiting-list prioritization management systems in Canada, as elsewhere.

## Background

The organization and management of waiting lists continue to be of significant interest to patients, care-providers, system-managers, and policy makers, particularly among publicly-funded health care systems where access constraints to specialized medical procedures exists [1-5]. In most jurisdictions, the prioritization of patients in the queue occurs implicitly, based on clinical necessity, but without predefined or standardized rules.

Canada's universal publicly-funded health care system is designed to ensure that access to medical care is delivered equitably to all of its citizens. In Ontario, waiting list for coronary artery bypass (CABG) surgery is among the first examples worldwide of a triage system based on explicit criteria designed to capture clinical urgency but exclusionary of age, comorbidity, and socioeconomic status[6]. Yet, non-clinical factors, such as patients' age and work status may influence how individuals are prioritized in the CABG queue [7-9]. In other international jurisdictions, prioritization and utilization criteria have adopted non-clinical factors, such as age, into explicit waiting-list management systems to reflect common societal values and to help improve the consistency and transparency of the triage process[5,10]. However, no study has explored the perspectives of actual patients in the queue to determine how they would prioritize others relative to themselves based on varying clinical and non-clinical parameters.

Accordingly, the objective of this study was to examine the extent to which clinical and non-clinical factors impacted on how patients would prioritize others relative to themselves in the coronary artery bypass surgical queue in Ontario, Canada. Using a series of scenarios, we specifically examined clinical urgency, age, and social independence because these are factors which have been shown by others to be important among stakeholders in wait list management [5,6,9,10]. Ontario provides an ideal test jurisdiction in which to examine public attitudes to prioritization for three reasons:

First, at the time we conducted our study, Ontario was the only province in Canada which incorporated an explicit waiting-list management system, based entirely on markers reflective of clinical necessity. Second, Canada is among the few countries worldwide that generally prohibit parallel private access. While there is intense debate within each of its provincial jurisdictions over the future of Medicare [11,12], there has been little, if any societal debate with regards to the relative merits and pitfalls associated with the selective integration of social-factors into public-funding models, despite the fact that such factors have already been shown to influence cardiac service delivery in Ontario[7,13]. We hypothesized that patients in the CABG surgical queue would value Canada's universal Medicare principals and prioritize themselves, and others based on clinical acuity, but would also be willing to cede their own place in the queue to others who were younger, socially independent and employed individuals [7,8].

## Methods

### Context

Ontario has the largest population of any province in Canada. Patients awaiting bypass surgery are assigned an urgency score based on symptoms, angiographic findings, left ventricular function, and non-invasive tests for ischemia. Individual urgency scores are then linked to a maximum recommended waiting time for surgery. A panel of cardiovascular specialists developed the urgency score in 1990 [6], and in 1995, it was validated in a population-based study [14]. Sunnybrook Health Sciences Centre (SHSC) is an academic, tertiary care hospital in Ontario, Canada. At the time of this study, the average volume of coronary artery bypass surgery at SHSC was 90 cases per month, approximately 10% of the total cases in Ontario [15].

### Sample

Our study sample comprised only a small subset of the total coronary artery bypass surgical case-load volume at SHSC, and was confined to ambulatory-care patients accepted for coronary artery bypass grafting surgery who attended their outpatient preoperative assessment class (approximately 2–4 weeks prior to the patient's scheduled date for surgery) between June 2003 and February 2004. Two registered nurses approached consecutive patients and distributed the survey with a standardized preamble as introduction. Patients who were awaiting concomitant valve surgery and non-English speaking patients were excluded from the study sample. Study participation required written consent. The study was approved by the research ethics board at SHSC.

### Survey

The survey included collection of baseline characteristics, including age, sex, education, clinical urgency as derived using a validated index (herein termed the CCN urgency rating scale), and a series of hypothetical patient scenarios (Table 1). In each scenario, the respondent was asked to rank where they would place the hypothetical patient in the queue for CABG surgery compared to them. For example, in Scenario A, respondents were asked: "Patient A is at home awaiting bypass surgery for an identical surgical procedure to you. They have waited an identical length of time, and are experiencing identical heart symptoms to your own. The patient is also your age and has similar medical problems. Please rank where you think Patient A should be on the waiting list compared to you." The rankings were distributed along a time scale relative to the survey respondents' place in the queue for CABG surgery, ranging from "very far behind you" to "very far ahead of you". Scenario A2 was identical except the hypothetical patient had increasing angina and was then admitted to hospital.

**Table 1:** Patient Scenarios Presented In Survey.

**Scenario**	**Description**
A	Exactly the same as respondent
A2	Admitted to hospital with increasing angina
	
B	85 years old
C	85 years old, mild dementia, living at home with home care support
D	85 years old, severe dementia, living in nursing home
	
E	35 years old
F	35 years old, married with 4 children and only income earner for family
G	35 years old, unmarried, unemployed on welfare
	
H	Donated $5 million dollars to the hospital foundation

Scenario B concerned a patient who was identical to the respondent other than being 85 years of age. Scenario C was identical except the patient had mild dementia and required home care support. Scenario D was identical to Scenario B, except the patient had severe dementia, and was living in a nursing home.

Scenario E concerned a patient who was identical to the respondent other than being 35 years of age. Scenario F was identical except the patient was married with four children and was the sole income provider for their family. Scenario G was identical to Scenario E, except the patient was unmarried, and unemployed on welfare support.

Scenario H concerned a patient who was identical to the respondent other than having donated $5 million to the hospital foundation. Two additional questions were then asked about patient's perspectives to pay out-of-pocket in order to incur shorter waiting times in the bypass surgical queue. Using a five item ordinal scale whose responses ranged from 'strongly agree' to 'strongly disagree', patients were asked first, whether those who could afford to pay for bypass surgery should be able to receive their surgery quicker than those who could not afford to pay, and second, whether they themselves would be willing to pay money out of pocket to avoid having to incur any waiting list for bypass surgery? Responses were then re-categorized into three groups for comparison: strongly agree/agree vs. uncertain vs. disagree/strongly disagree.

### Analytic techniques

Responses to the scenarios A through H were assigned numerical values from -2 to +2 with a mean of zero representing a rank of "same place" in the queue as respondent. Wilcoxon signed rank statistic was used to compare the response for each scenario to a mean of zero. Bonferroni correction was used for the nine sequential scenarios to adjust for multiple comparisons, with statistical significance set at a p-value of 0.005 or less.

A sensitivity analysis adjusted for the respondent's baseline positioning of patients in the queue, by examining the extent to which the placement of others in the queue explored in scenarios B to G compared with his/her baseline response to scenario A. To further help disentangle the effects of age from social dependency when placing others relative to themselves in the queue, we undertook a series of pairwise comparisons to evaluate the sequential impact of increasing social support needs within each of two age extremes, with scenarios B through D examining increasing social dependency within a hypothetical 85 year old patient, while scenarios E through G examining increasing social dependency within a hypothetical 35 year old patient. Finally, subgroup analyses examined whether responses significant varied according to patient gender, age, education, and clinical urgency – the latter three stratified around the sample's median for their respective subgroups.

## Results

Surveys were distributed to 91 consecutive English-speaking patients. There were 59 respondents, and 31 non-respondents [no consent (3), not returned (18), declined (11)], giving an eligible response rate of 65%. When compared to all out-patients undergoing bypass surgery at SHSC and in the province over the identical study period, mean ages were similar (mean age in years +/- SD: 65.1 +/- 8.8 vs. 66.0 +/- 9.5 +/- vs. 65.3 +/- 9.8; survey respondents vs. SHSC vs. Ontario). However, the proportion of females relative to males, and the proportion of elective relative to semi-urgent and urgent were modestly higher among survey participants than those comprising Sunnybrook's and the province's out-patient coronary artery bypass surgical waiting-list population.

Table 2 illustrates the baseline characteristics of the study sample. The mean age was 65, and the majority of patients were male. Half of the patients had high school education or less. The majority of patients were employed or retired, with only 1.6% not working secondary to disability. No patients were ranked as emergent or urgent on the CCN Urgency rating scale. 20% were ranked as semi-urgent, and 66% as elective. The median time spent in the queue prior to survey administration was 8 weeks (IQR: 6–12 weeks).

**Table 2:** Baseline characteristics of survey respondents

**Characteristic**	**Survey Respondents (n = 59)**
**Age (mean ± SD)**	65.05 ± 8.82
	
**Gender (n, %)**	
Male	40 (67.8)
Female	19 (32.2)
	
**Education (n, %)**	
High school or less	28 (47.5)
Post-Secondary	28 (47.5)
Missing	3 (5.1)
	
CCN Urgency Rating (n, %)*	
Emergency/Urgent (0–14 days)	0 (0)
Semi-Urgent (15–42 days)	12 (20.3)
Elective (43–180 days)	39 (66.1)
Missing	8 (13.6)
	
**Weeks spent in the queue (median, IQR)**	8 (6, 12)

* CCN Urgency Rating extrapolated from total time patient had been on waiting list to date in addition to expected wait list until scheduled procedure

SD = standard deviation

**Figure 1:**
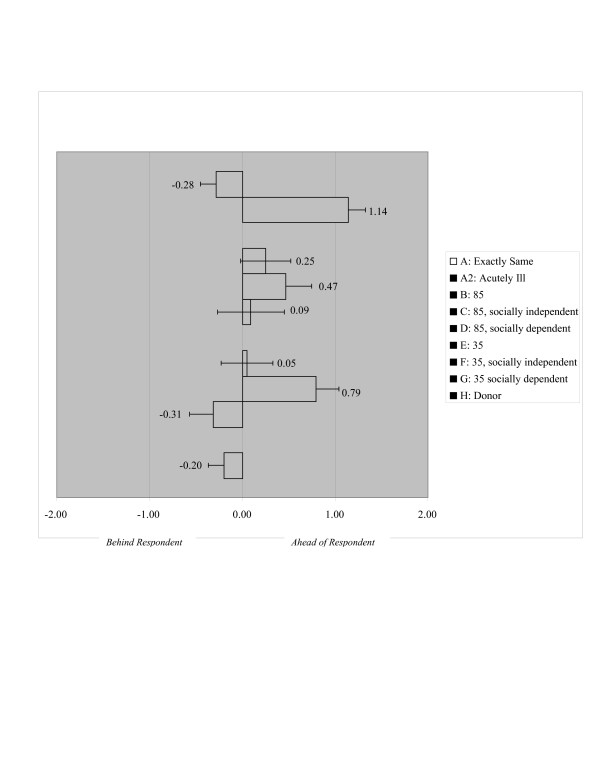
Unadjusted survey responses for scenarios A-H, compared to survey respondent's place in queue.

Table 3 and Figure 1 illustrate the survey responses to the Scenarios A through H. The responses are compared to a mean of zero, signifying that any rankings above zero indicate the respondent would place the hypothetical patient ahead of himself or herself in the queue, while rankings below zero indicate the respondent would place the hypothetical patient behind them in the queue. At baseline, respondents put themselves ahead in the queue even when compared with a patient who was exactly the same as themselves (Scenario A). When faced with a patient who has become acutely ill (Scenario A2), respondents placed this patient significantly ahead of themselves in the queue.

**Table 3:** Survey Responses for Scenarios A-H, relative to the respondent's own position in the queue

Scenario*	Mean difference in the rank score relative to respondent's own position**	**SD**	**signed rank statistic**	signed rank p-value***
A: exactly the same as respondent	-0.28	0.64	-100.0	0.0011
A2: admitted to hospital with increasing angina	1.14	0.74	654.0	<.0001
B: 85 yrs old	0.25	1.02	141.5	0.0718
C: 85 yrs old, mild dementia, living at home with home care support	0.47	1.06	247.0	0.0015
D: 85 yrs old, severe dementia, living in nursing home	0.09	1.35	55.0	0.5134
E: 35 yrs old	0.05	1.06	31.0	0.6137
F: 35 yrs old, married with 4 children and only income earner for family	0.79	0.96	399.0	<.0001
G: 35 yrs old, unmarried, unemployed on welfare	-0.31	0.99	-162.0	0.0218
H: donated $5 million dollars to the hospital foundation	-0.20	0.64	-71.5	0.0245

*answer scale: 0 = very far behind you, 1 = a little bit behind you, 2 = your place, 3 = a little bit ahead of you, 4 = very far ahead of you

**ranges from -4 to 4; a mean of 0 indicates that the survey respondent ranks the person in the same place as themself; values above zero indicate the survey respondent ranks the person ahead of themself in the queue, values below zero indicate the survey respondent rank the person behind themself in the queue

***tests whether mean is significantly different from 0. P-values have been corrected for multiple comparisons by Bonferroni adjustment;

statistical significance is p ≤ 0.005

SD = standard deviation

Scenario B, C and D represent a patient 85 years of age with increasing functional impairment. Advanced age itself had no significant effect on the respondents' ranking of a hypothetical patient in the queue. Respondents ranked the patient of advanced age with mild functional impairment slightly ahead of themselves in the queue. The tendency to place this older patient ahead in the queue was lost as the patient became more severely functionally impaired. Scenario E, F, and G represent a patient 35 years of age with varying social circumstances. Young age itself had no significant effect on the respondents' ranking of a hypothetical patient. Respondents ranked the young patient who is working full-time and supporting a family slightly ahead of themselves in the queue. The tendency to place this younger patient ahead in the queue was lost as the patient became more socially disadvantaged and dependent on welfare. Financial status had no significant effect on the respondents' ranking of a patient in the queue compared to themselves (Scenario H) (Table 3, figure 1).

In total, 96.4% of patients disagreed or strongly disagreed with the use of out-of-pocket payments to advance the position of others in the queue. While the majority of respondents (69.7% of respondents) did not support the use of out-of-pocket payments to self-advance their own place in the queue, there was greater uncertainty expressed in the use of out-of-pocket payment to advance their own position than such payments to advance the position of others in the queue (21.4% vs. 1.8%, respectively).

### Sensitivity and subgroup analyses

When Scenarios B to G were subdivided and examined within each of their respective age strata, social independence was increasingly valued particularly for young patients, whereby prioritized 35 year old sole family-income provider ahead of themselves in the queue, despite having otherwise identical clinical indications and severity as themselves (Table 4).

**Table 4:** Pairwise comparisons to questions which examine the importance of social and cognitive dependence within age strata

Scenario*	Mean difference in the rank score relative to respondent's own position**	**SD**	**signed rank statistic**	signed rank p-value***
An 85 year old who has the same clinical indication and severity as that of the respondent's, but with mild dementia who lives at home who receives support for groceries/cleaning ***vs***. an 85 year old patient who is otherwise identical to the respondent	0.25	0.91	53.5	0.05
An 85 year old who has the same clinical indication and severity as that of the respondent's, but with severe dementia, living in a nursing home with full nursing care ***vs***. an 85 year old patient who is otherwise similar to the respondent but with mild dementia who lives at home who receives support for groceries/cleaning	-0.39	1.11	-116.0	0.007
An 85 year old who has the same clinical indication and severity as that of the respondent's, but with severe dementia, living in a nursing home with full nursing care ***vs***. an 85 year old patient who is otherwise identical to the respondent	-0.14	1.21	-44.5	0.42
A 35 year old who has the same clinical indication and severity as that of the respondent's, but is married with 4 children, receiving average salary and is the family's sole income-provider ***vs***. a 35 year old is otherwise identical to the respondent	0.74	0.94	195.5	<0.0001
A 35 year old who has the same clinical indication and severity as that of the respondent's, but is unmarried, unemployed, on welfare ***vs***. a 35 year old who has the same clinical indication and severity as that of the respondent's, but is married with 4 children, receiving average salary and is the family's sole income-provider	-1.12	1.02	-370.5	<0.0001
A 35 year old who has the same clinical indication and severity as those of the respondents, but is unmarried, unemployed, on welfare ***vs***. a 35 year old is otherwise identical to the respondent	-0.39	0.94	-71.0	0.003

*answer scale: 0 = very far behind you, 1 = a little bit behind you, 2 = your place, 3 = a little bit ahead of you, 4 = very far ahead of you

**ranges from -4 to 4; a mean of 0 indicates that the survey respondent ranks the person in the same place as themself; values above zero indicate the survey respondent ranks the person ahead of themself in the queue, values below zero indicate the survey respondent rank the person behind themself in the queue

***tests whether mean is significantly different from 0. P-values have been corrected for multiple comparisons by Bonferroni adjustment;

statistical significance is p ≤ 0.005

SD = standard deviation

Subgroup analyses of the survey responses by subgroups of age, gender, education, and CCN urgency scale was performed (Table 5 and Table 6). There was no evidence that the respondents' own sociodemographic characteristics or illness severity impacted on their ranking of the hypothetical patients in the scenarios.

**Table 5:** Subgroup analysis of Survey Responses for Scenarios A-H by Subgroups of Age and Gender

	**Age**					**Gender**				
			
**Scenario**	**< 67 yrs old**		**>= 67 yrs old**		**Fisher's exact p-value**	**Male**		**Female**		**Fisher's exact p-value**
			
	Mean*	**SD**	Mean*	**SD**		Mean*	**SD**	Mean*	**SD**	
A: exactly the same as respondent	1.93	0.65	1.52	0.57	0.0367	1.78	0.62	1.61	0.70	0.1177
A2: admitted to hospital with increasing angina	3.29	0.71	3.00	0.74	0.2955	3.21	0.77	3.00	0.67	0.1328
B: 85 yrs old	2.21	1.13	2.28	0.92	0.3274	2.31	1.06	2.11	0.96	0.1719
C: 85 yrs old, mild dementia, living at home with home care support	2.46	0.92	2.47	1.20	0.6225	2.51	1.00	2.37	1.21	0.3738
D: 85 yrs old, severe dementia, living in nursing home	1.82	1.36	2.36	1.31	0.0662	2.31	1.30	1.59	1.37	0.2936
E: 35 yrs old	2.21	0.83	1.90	1.23	0.0360	1.95	1.05	2.28	1.07	0.8809
F: 35 yrs old, married with 4 children and only income earner for family	2.96	0.79	2.62	1.08	0.6340	2.69	1.00	3.00	0.84	0.9325
G: 35 yrs old, unmarried, unemployed on welfare	1.89	0.92	1.50	1.04	0.3554	1.64	0.93	1.79	1.13	0.6895
H: donated $5 million dollars to the hospital foundation	1.79	0.57	1.82	0.72	0.3069	1.79	0.62	1.83	0.71	0.7207

*answer scale: 0 = very far behind you, 1 = a little bit behind you, 2 = your place, 3 = a little bit ahead of you, 4 = very far ahead of you SD = standard deviation

CCN = Cardiac Care Network (of Ontario)

**Table 6:** Subgroup Analysis of Survey Responses for Scenarios A-H by Subgroups of Education and Illness Severity

	**Education**					**CCN Urgency Scale**				
	
**Scenario**	**High school or less**		**Post-secondary**		**Fisher's exact p-value**	**Emergent/Semi-Urgent**		**Elective**		**Fisher's exact p-value**
				
	Mean*	**SD**	Mean*	**SD**		Mean*	**SD**	Mean*	**SD**	
A: exactly the same as respondent	1.63	0.74	1.71	0.46	0.0943	1.67	0.49	1.75	0.67	1.0000
A2: admitted to hospital with increasing angina	3.11	0.75	3.18	0.72	1.0000	3.17	0.71	3.13	0.75	0.4726
B: 85 yrs old	2.54	0.86	1.96	1.10	0.0889	2.06	0.80	2.29	1.07	0.4420
C: 85 yrs old, mild dementia, living at home with home care support	2.59	1.08	2.29	1.05	0.8172	2.39	0.98	2.44	0.98	0.9483
D: 86 yrs old, severe dementia, living in nursing home	2.08	1.41	2.07	1.27	0.8605	1.89	1.18	2.17	1.32	0.8335
E: 35 yrs old	2.15	1.08	1.96	1.00	0.6388	2.17	0.86	2.06	1.15	0.2863
F: 35 yrs old, married with 4 children and only income earner for family	3.04	0.82	2.64	1.03	0.7009	3.06	0.80	2.71	1.01	0.8935
G: 35 yrs old, unmarried, unemployed on welfare	1.89	1.12	1.54	0.79	0.2820	1.83	0.71	1.81	1.06	0.4569
H: donated $5 million dollars to the hospital foundation	1.69	0.62	1.85	0.66	0.7337	2.00	0.50	1.84	0.69	0.2157

*answer scale: 0 = very far behind you, 1 = a little bit behind you, 2 = your place, 3 = a little bit ahead of you, 4 = very far ahead of you

SD = standard deviation

CCN = Cardiac Care Network (of Ontario)

## Discussion

Our study determined that respondents placed themselves marginally ahead of others who were of similar clinical acuity in the bypass surgical queue. While age itself did not significantly impact waiting list prioritization perspectives, respondents positively valued social and/or cognitive independence. For example, a hypothetical elderly patient with mild functional impairment living independently in the community received similar, if not more urgent priority than a respondent's own position in the queue. However, such elderly patients were given significantly lower priority if cognitive functional decline was present. Respondents also acknowledged a willingness to cede their place in the queue for a young sole-income earner with family dependants. However, such preferential access perspectives were lost when we explored an otherwise similar hypothetical patient, but who was now unemployed and social-service dependent. Neither financial status nor personal income contributions to the institution had a significant impact on the respondents' waiting-list prioritization perspectives. Moreover, the vast majority of respondents advocated against self-payment options to expedited service access.

In Ontario, the triage system for coronary artery bypass (CABG) surgery is based on disease severity, which is linked to explicit urgency scores and maximum recommended waiting time. The exclusion of age and socioeconomic factors from the determination of urgency rating scales was designed by intent to ensure that queuing priority was conducted in a transparent and equitable fashion based entirely on clinical necessity [6]. While reflective of common Canadian social values [5,9,16], few studies have explored whether such perspectives are apply to key stakeholders [7], particularly those patients currently experiencing delays in the queue. Our findings reaffirm the importance of clinical acuity and necessity as central factors which should govern the waiting-list priority of patients in the bypass surgical queue.

Notwithstanding the importance of clinical necessity, patients in the bypass surgery queue placed young sole-income family-providers ahead of themselves in the queue, and conversely positioned welfare dependents significantly behind. The valuation of social independence is consistent with the views of other stakeholders in Canada, and elsewhere [8,9]. However, unlike other studies which examined the perspectives of public and physician stakeholders [8,9], age itself did not significantly impact on perceptions of waiting list priority. Such inconsistency may have been attributable to the fact that age may serve as a surrogate for other social and cognitive attributes – both of which impacted on patients' waiting-list prioritization preferences.

Wait lists for medical care have become foremost in the public eye over recent years. The Canadian Medical Association commissioned a poll of the general adult Canadian public on attitudes towards wait lists during February 2004 [17]. The majority of those surveyed agreed that triage criteria should include the risk of death (69%) and the pain of the patient (51%). Fewer Canadians believed that triage criteria should include age (22%), ability to work (22%), household responsibilities (19%), or the ability to pay (14%). While the majority of respondents in our study advocated against hospital donors or the use of out-of-pocket payments for queue advancement, the preferential access perceptions of social independence may justify further societal debate over the merits of including selected non-clinical factors into explicit waiting-list and utilization decision-making criteria.

Our study has several important limitations.

First, our sample size was small, and we had limited statistical power to detect potentially clinically meaningful subgroup differences in responses. Moreover, the clinical importance of statistical differences in self-rated priority relative to others is unknown, and beyond the scope of our study. Therefore, our results require validation and should only be interpreted as hypotheses generating.

Second, all patients were awaiting one service from a single institution. It is possible that responses may have varied across institutions. Sunnybrook accounts for approximately 10% of the total volume of bypass surgery in the province, and similar urgency rating scales are used for waiting list prioritization in all Ontario tertiary institutions. Therefore, we believe that our findings would have been similar, had we expanded the sample size and the numbers of institutions in our study. Indeed, in another recent study examining the attitudes of 2,256 acute myocardial infarction survivors toward the use of out-of-pocket payment to improve access to cardiovascular services in Ontario, only 20% of respondents advocated in favor of a parallel private stream to advance patients priority placement and/or selection of treatments, thereby further reaffirming the findings of our study.

Third, deaths resulting from delays for bypass surgery have been well documented and publicized, and may have resulted in patients placing greater importance on clinical acuity than they otherwise would have had they been in the queue awaiting services associated exclusively with morbidity outcomes [14].

Fourth, our study did not examine how time in the queue might have varied patients' prioritization perspectives. One might hypothesize that queuing preferences may shift or evolve with the amount of time waiting in the queue. Nonetheless, study respondents had already experienced significant time in the queue (median duration of eight weeks), with relatively little variation across respondents.

Finally, we provided no information on how the prioritization of others relative to themselves might have impacted on their likelihood of experiencing an adverse event in the queue. It is possible that a patient's likelihood to cede their place relative to others will vary depending upon his/her perception of the additional risks and consequences. While each patient was likely informed about the risk of adverse events in the bypass surgical queue, such perceptions may have variably affected the survey responses.

In conclusion, patients on waiting-lists for coronary artery bypass surgery placed themselves marginally ahead of others of similar acuity, but acknowledge the importance of clinical acuity by positioning others who were of greater urgency ahead of themselves in the queue. The valuation of social independence particularly among young sole-family income providers is consistent with other findings and may justify further debate over the inclusion of selected non-clinical criteria in explicit waiting-list priority management systems.

## Authors' contributions

KS participated in the survey development, design, and coordination and helped to draft the manuscript. AC performed statistical analyses for the manuscript. DAA conceived of the study, and participated in the survey development, design, supervision and helped to draft and revised the manuscript. All authors read and approved the final manuscript.

## Pre-publication history

The pre-publication history for this paper can be accessed here:


